# Exploring Associations Between Social Risk Factors and Physical Frailty in Heart Failure: A Cross-Sectional Analysis

**DOI:** 10.1016/j.yjcafi.2026.01.009

**Published:** 2026-07-15

**Authors:** QUIN E. DENFELD, CHRISTOPHER V. CHIEN, ZIJIAO WANG, SHIRIN O. HIATT, MARY ROBERTS DAVIS, CHRISTOPHER S. LEE

**Affiliations:** 1 Oregon Health & Science University School of Nursing, Portland, Oregon; 2 Oregon Health & Science University Knight Cardiovascular Institute Portland, Portland, Oregon; 3 Boston College William F. Connell School of Nursing, Chestnut Hill, Massachusetts; 4 Division of Scientific Research and Innovation in Emergency Medical Service, Department of Emergency Medical Service, Faculty of Nursing and Midwifery, Wroclaw Medical University, Wroclaw, Poland

## Introduction

Physical frailty affects approximately half of patients with heart failure (HF) and portends worse outcomes.^1^ Biological mechanisms (eg, inflammation) and clinical manifestations (eg, comorbidities) have been linked with physical frailty in HF,^2^ including sex differences.^3^ Although social risk factors of frailty (eg, education, social support^4^) have been identified among older adults, these have not been well characterized in HF. Social risk factors may influence the development and manifestation of frailty, particularly among women. The purpose of this study was to (1) identify associations between social risk factors and physical frailty among adults with HF and (2) explore the influence of sex on these associations.

## Methods

This is a secondary analysis of combined data from 2 studies that focused on physical frailty in HF and enrolled patients with New York Heart Association (NYHA) functional classification I-IV HF from a single academic medical center.^3,5^ Inclusion criteria were similar: age ≥21 years of age and ability to read and comprehend fifth-grade English. Both studies had similar exclusion criteria: documented major cognitive impairment or active psychosis, previous heart transplantation or mechanical circulatory support, major hearing dysfunction, or otherwise unable to complete the requirements of the study. Both studies were approved by the institutional review board, and written informed consent was obtained, including storing data for future analyses.

### Measurement

#### Social Risk Factors and Clinical Data.

We applied the World Health Organization model on social determinants of health (ie, sex, race/ethnicity, highest level of education, financial status, and employment status).^6^ Also, given the importance of relationships and community in predicting frailty among older adults,^4^ we examined marital/partnered status and having someone to confide in (proxy for loneliness). These data were collected using sociodemographic questionnaires. We performed a medical record review to collect data on HF history, etiology, NYHA functional class, and comorbidities.

#### Physical Frailty.

Using the Frailty Phenotype Criteria, we assessed the 5 criteria of physical frailty: shrinking, weakness, slowness, physical exhaustion, and low physical activity. Our measurement of physical frailty has been previously described.^3,5^ Each participant was classified as either “nonphysically frail” (0–2 criteria met) or “physically frail” (≥3 criteria met).

### Analysis

Descriptive and comparative statistics were used to describe the sample and quantify differences between participants who were identified as physically frail and those not identified as physically frail. To identify predictors of physical frailty, we used multivariate logistic regression models. To quantify the additional contribution of social risk factors in predicting physical frailty, we used a hierarchical multivariate logistic regression model in which the first block included clinical risk factors and the second block included social risk factors. We explored the influence of sex on associations between each factor and physical frailty using interaction testing and predictive margins plotting. All analyses were conducted in Stata MP 18.0 (College Station, TX).

## Results

The combined sample (n= 160) was, on average, 61.7 years old, 44% female, and mostly NYHA class III/IV (Table 1). Physical frailty was present in 44% of the sample. In unadjusted comparisons, the physically frail group was significantly older, more female, had more comorbidities, and contained a greater proportion of those who were unemployed or retired and did not have enough money to make ends meet.

**Fig. 1.**
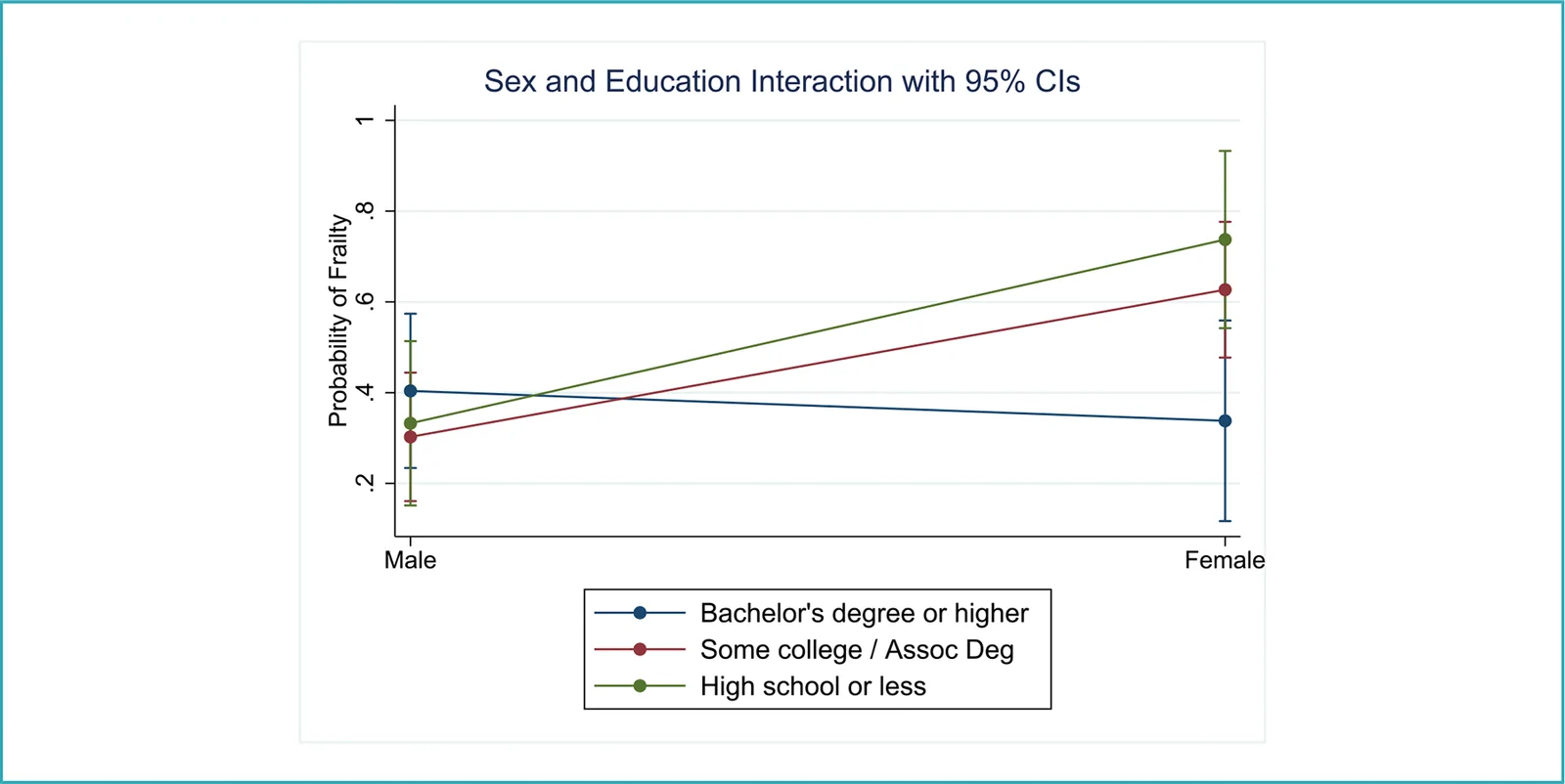
Interaction of sex and educational level in predicting physical frailty. The association between educational level and physical frailty depends on whether someone is a female or male. For women, those who are less educated have a greater likelihood of being physically frail compared with those more educated. For men, their educational level does not appear to influence the likelihood of being physically frail or not.

In the multivariate model (Table 2), female sex and unemployment or retirement were associated with greater odds of physical frailty compared with men and current employment, respectively. A hierarchical logistic regression model showed that the block of social risk factors significantly predicted physical frailty beyond clinical risk factors (Wald *χ*^2^ = 24.28, *P* = .0021; Δ pseudo R^2^ = 14.2%). The only interaction statistically significant was sex by education. Specifically, there was a gradient of greater probability of physical frailty with lower levels of education for women but not for men (interaction *P* < .05; Fig. 1).

## Discussion

We found that social risk factors meaningfully predicted physical frailty beyond clinical risk factors in HF, especially for women and those unemployed or retired. We also observed that the association between level of education and physical frailty differed by sex, with lower educational attainment associated with a greater probability of physical frailty in women relative to men with the same education. These findings support incorporating social risk factors and sex-specific interactions when addressing physical frailty in HF.

Studies among older adults have demonstrated that social factors are linked with frailty, consistent with what we have observed. Specifically, lower educational attainment and increased financial strain have been linked to frailty among older adults.^4^ We found that retirement or unemployment was a strong predictor of physical frailty, suggesting that employment status change may be a sentinel event in adults with HF. Increased financial strain from loss of employment may affect medication adherence and self-care behaviors, undermining the management of HF and leading to physical frailty.^7^ Also, leaving work affects overall daily structure, reducing physical activity^8^ and increasing isolation and loneliness.^9^

We found that with lower education levels, women have a greater likelihood of physical frailty than men with the same education; however, the gap narrows with higher education. This finding aligns with evidence that lower educational attainment is linked with greater frailty among women.^10^ The mechanisms underpinning this finding could relate to structural influences (eg, access to resources and social networks) that more strongly influence this pathway in women.

There are limitations and future research to consider. As this was a single center cross-sectional study with a predominantly Non-Hispanic White sample, longitudinal, multicenter designs with broader geographic and racial/ethnic representation are needed. Also, we were unable to analyze more granular social risk factors (eg, formal vs informal support, employment type and duration), which should be explored in future research. Finally, there is a need to develop and test interventions targeted to at-risk groups, such as those who have an employment status change or women with lower education.

## Conclusion

Social risk factors, individually and in combination, are potential significant contributors to physical frailty in HF. Retirement or unemployment was a notable predictor, and female sex remains a strong predictor of physical frailty in HF. Future work is needed to understand the social-environmental context in which physical frailty develops and worsens in HF.

## Figures and Tables

**Table 1:** Characteristics of the sample and by level of physical frailty

	Total (n = 160)	Nonphysically Frail (n = 89)*	Physically Frail (n = 71)	*P* Value

Patient and social characteristics				
Age, y	61.7 ± 14.6	59.7 ± 15.3	64.3 ± 13.3	.040
Female	71 (44%)	29 (33%)	42 (59%)	.001
Non-Hispanic White	132 (83%)	75 (84%)	57 (80%)	.510
Retired or unemployed	124 (78%)	60 (67%)	64 (90%)	.001
Education				.107
High school or less	41 (26%)	18 (20%)	23 (32%)	
Some college/associate's degree	69 (43%)	38 (43%)	31 (44%)	
Bachelor's degree or higher	50 (31%)	33 (37%)	17 (24%)	
Do not have enough to make ends meet	43 (27%)	18 (20%)	25 (35%)	.034
Single, divorced, or widowed	66 (41%)	32 (36%)	34 (48%)	.128
Do not have someone in whom to confide	10 (6%)	7 (8%)	3 (4%)	.514
Clinical characteristics				
Body mass index, kg/m^2^	31.0 ± 8.1	29.8 ± 7.5	32.6 ± 8.6	.030
Charlson Comorbidity Index (weighted)	3 [2–4]	2 [1–3]	3 [2–4]	.021
Stage 3 chronic kidney disease	39 (24%)	16 (18%)	23 (32%)	.035
Type 2 diabetes	59 (37%)	23 (26%)	36 (51%)	.001
Heart failure characteristics				
New York Heart Association functional class III/IV	99 (62%)	48 (54%)	51 (72%)	.021
Non-ischemic etiology	115 (72%)	66 (74%)	49 (69%)	.472
Left ventricular ejection fraction, %	37.7 ± 16.5	36.1 ± 15.0	39.8 ± 18.0	.165

Values are mean ± standard deviation, median [interquartile range], or n (%).

* Nonphysically frail includes both nonfrail and prefrail.

**Table 2:** Clinical and social predictors of physical frailty

	OR [95% CI]	*P* Value

Age, y	1.02 [0.99–1.05]	.199
NYHA class III/IV	2.06 [0.90–4.73]	.089
Charlson Comorbidity Index	1.23 [0.99–1.53]	.057
Female sex (vs male)	3.67 [1.62–8.33]	.002
Minority racial/ethnic group (vs non-Hispanic White)	2.38 [0.83–6.78]	.105
Education		
High school or less	1.90 [0.67–5.38]	.226
Some college/associate's degree	1.32 [0.67–5.38]	.562
College degree or higher	ref	
Do not have enough to make ends meet (vs have enough or more than enough)	1.87 [0.79–4.47]	.156
Unemployed or retired (vs employed part- or full-time)	3.86 [1.38–10.80]	.010
Single, divorced, or widowed (vs married or partnered)	1.50 [0.67–3.32]	.321
Do not have someone in whom to confide (vs have someone in whom to confide)	2.95 [0.47–18.76]	.251

Model pseudo R^2^ / area under the ROC curve: 20.3% / 0.784.

CI, confidence interval; NYHA, New York Heart Association; OR, odds ratio; ROC, receiver operating characteristic.
